# Supplementary material for: The prevalence of hypertension and hypertension control among married Namibian couples
Source: PLoS One. 2023 Aug 10;18(8):e0289788. doi: 10.1371/journal.pone.0289788 (PMC10414666; doi:10.1371/journal.pone.0289788)
Supplement: Attachment — Submitted filename: Comments to HTN in Namibia.docx [file pone.0289788.s001.docx]

- Thank you the editor in chief, for providing this golden opportunity to me to review the interesting manuscript. This paper used very strong multilevel model to assess the prevalence, and control of HTN in Namibian couples. The researcher also identified those factors which had association with the prevalence and control of HTN in couples. There are very interesting finds which can play a great role in alleviating the increasing burden of NCD. However, to assure its contribution for readers, still it needs a great work. For this matter I tried to put my concerns here below headed as minor and major comments. I hope the author will cover all points and make the manuscript sounder. Looking for the modified document!!
- Minor Comments:
- Line 35-41: In abstract, the result section does not include all relevant findings in line with the topic of the study.
- Line 88-96: Citation has to be put.
- Line 99-102: Why the research question was mixed with the Methods? It has to be separated!
- Line 169 (Fig 1): Mention it in the method section.
- Line 171: What type of weighing was used? Why?
- Line 171-184: The citation of table missed.
- Line 171: The appropriate heading needs to be given for the first objective which was “The prevalence of HTN in couples” and you need to compare both groups too with respect to the outcome variable.
- Line 171-177: You mentioned as the males and females experienced the variability of HTN level across the categories of individual characteristics. But, there is no any comparison statistics in table 1 that put in the narration. It would be better to put the actual P-values in the table.
- Table 1: How the variables like age, BMI, wealth index were measured? The unit? Additionally mention them in method section. Better to make the table topic more self-explanatory! When? Where?
- Line 201: You defined uncontrolled HTN as those who were either unaware of their hypertension status or those who were aware but not controlled. Here both partners were unaware of their hypertension in 37.7% of couples. What about those who were aware but not controlled? And also in line 203 both partners were in control of their hypertension in only 8.31% of hypertensive couples. Which means those with uncontrolled HTN would be about 91.7%? What about 37.7%?
- Line 189: Individuals? Male? Female?
- Line 229: The association was simply marginal, you discusses as if they had association. What??
- Line 239: In contrast? Two similar issues are compared. Why you mentioned as a contrast?
- Line 241: Regarding residency, rural or urban category was obtained as a factor? Correct it.
- Line 257: 3.67?
- Major comments:
- The author operationalized the “HTN control” in line 118-119. However, how those who were aware but not controlled were identified in NDHS survey? It has to be mentioned in a clear way. Generally, the way how an author categorized either controlled or uncontrolled HTN is not clear. The survey was not facility based and it was a snapshot. So, how confident is the author to measure and report the individuals’ HTN control status.
- Even though secondary data was used, the author has to explain the following points in method part deeply: The study setting? The setting characteristics?, which population data set was used in this analysis?, how many of them fulfilled the inclusion criteria, how many of them were removed/dropped? (492 couples for HTN prevalence Vs 121 for HTN control), what study design was applied ?, how the study subjects were recruited? (all stages of sampling need to be explained in detail), what type of weighting was applied? And why?, how the data quality was assured in NDHS?, how missed variable were managed?, why a Multilevel LR model was applied?, how much was the cluster correlation level (within-cluster correlation)?, how you measured?, at what level of intra-cluster correlation the multilevel analysis is recommended?, what individual and community level factors were considered, how you assessed your model fitness? (The steps of model building have to be explained clearly). Generally, the method section is poor in mentioning above issues. Hence, the authors must incorporate these points seriously.
- Discussion need to be sequenced based on the order of objectives of this study.
- The discussion is shallow especially for the factors obtained for the HTN. The possible explanations for each variable were not discussed deeply. So, re-write it.
- In discussion, you only tried to discuss those variables for females HTN. What about those factors for males’ HTN?
- In conclusion section, the authors have to make sure that all mentioned recommendations considered all identified factors.
- Did the researcher answer the research question? Which was the couple’s concordance in HTN prevalence and HTN control? I do not think so. How can we measure either the concordance exists or not? For me the researcher simply assessed the prevalence of HTN and associated factors for both sexes (males and females), and even though I do not agreed with the measurement of the variable HTN control status, the control status and factors were also assessed. I need clear explanation on these issue, and the author has to make the findings of this study more easily readable for the readers of this document.
- The topics for tables and figures have to be made self-explanatory and the fig. topic needs to be put at the appropriate place, preferably at the bottom of figures.
- For web page references, the URL, Access date and citation dates have to be incorporated.
- English editing is also highly needed.
