# Supplementary material for: The prevalence of hypertension and hypertension control among married Namibian couples
Source: PLoS One. 2023 Aug 10;18(8):e0289788. doi: 10.1371/journal.pone.0289788 (PMC10414666; doi:10.1371/journal.pone.0289788)
Supplement: Attachment — Submitted filename: Revised Response to Reviewers 190723_AW.docx [file pone.0289788.s003.docx]

July 19^th^ 2023

Response to Reviewers

Dear PLOS ONE editor and reviewers,

Thank you for taking the time to read and review our revised submission ‘The Prevalence of Hypertension and Hypertension Control Among Married Namibian Couples’. We are pleased that the reviewers felt all their comments have been addressed. We have addressed each of the editor comments and a point-by-point response in italics is included below.

**Additional Editor Comments:**

It is of great pleasure that the authors have thoroughly addressed reviewer's comments in their revised submission.

Just a minor comment:

1. The authors should identify specific confounding variables than putting an equivalence between hypertension risk factors versus confounding factors.
   **Authors’ response:** *We kept all significant factors (age, obesity, education, diabetes status, current smoking status, household wealth and residence) in the models as an indicator that they are hypertension risk factors. We did not formally assess whether they were confounders (changing the association of interest), therefore we have reviewed our references to potential confounders and amended the text for clarity.*
2. Also add who collected the data and efforts taken to maintain data quality.
   **Authors’ response:** *As this was secondary analysis of the Namibian DHS we have included additional detail regarding data collection and quality assurance from the DHS survey final report, line 73-75.*
3. Does the definition for obesity line 145 inclusive of BMI=30?
   **Authors’ response:** *The definition of obesity has been corrected to show inclusive of BMI=30, i.e. BMI≥30 (line 147).*
4. In figure 1, exclusion of 605 couples is from 1249 couples not from the 644 couples. as it currently stands, the exclusion seems from the 644 couples, and this should get corrected.
   **Authors’ response:** *The format of figure one has been corrected to clearly show the point at which exclusions took place.*

Thank you again for all your suggestions, we believe the changes have strengthened our paper.

Yours sincerely,

Alice Weare
